# Prognostic value of p53 and Ki67 expression in fiberoptic bronchial biopsies of patients with non small cell lung cancer

**DOI:** 10.1186/2049-6958-7-29

**Published:** 2012-09-14

**Authors:** Nicola Ciancio, Maria Grazia Galasso, Raffaele Campisi, Laura Bivona, Marcello Migliore, Giuseppe U Di Maria

**Affiliations:** 1 Pneumology Unit, University of Catania, Vittorio Emanuele Hospital, Catania, Italy; 2 Pathology Department, Garibaldi Hospital, Catania, Italy; 3 School of Specialization in Respiratory Diseases, University of Catania, Catania, Italy; 4 School of Specialization in Thoracic Surgery, University of Catania, Catania, Italy

## Abstract

**Background:**

Overexpression of the tumor suppressor gene p53 and the marker for cellular proliferation Ki67 in open lung biopsies are indicated as predictor factors of survival of patients with lung cancer. However, the prognostic value of p53 and Ki67 in fiberoptic bronchial biopsies (FBB) has not been fully investigated. We evaluated p53 and Ki67 immunostaining in FBB from 19 with Non Small-Cell Lung Cancer (NSCLC: 12 adenocarcinomas, 5 squamous cell carcinomas and 2 NSCLC-NOS).

**Methods:**

FBB specimens were fixed in formalin, embedded in paraffin, and immunostained using anti-p53 and anti-Ki67 antibodies. Slides were reviewed by two independent observers and classified as positive (+ve) when the number of cells with stained nuclei exceeded 15% for p53 or when >25% positive cells were observed throughout each section for Ki67.

**Results:**

Positive (+ve) immunostaining was found in 9 patients for p53 (47.37%) and 8 patients for Ki67 (42.10%). We examined overall survival curves of the patients with Mantel's logrank test, both p53 -ve and Ki67 -ve patients had significantly higher survival rates than p53 + ve (p < 0.005) and Ki67 + ve (p < 0,0001), respectively.

**Conclusion:**

This study suggests that negative immunostaining of fiberoptic bronchial biopsies for p53 and Ki67 could represent a better prognostic factor for patients with NSCLC.

## Background

Lung cancer accounts for the most cancer related deaths in both men and women. Cigarette smoking is by far the most important risk factor for lung cancer. Risk increases with quantity and duration of cigarette consumption. The 1-year relative survival for lung cancer increased up to 40% in the last ten years, largely due to improvements in surgical techniques and combined therapies. However, the 5-year survival rate for all stages combined is only 15%. Despite, the 5-year survival rate is 50% for cases detected when the disease is still localized, but only 16% when lung cancers are diagnosed out of this early stage, screening for early/localized lung cancer detection has not yet been proven to reduce mortality.

Some independent prognostic factors have been suggested for predicting survival and helping in the management of patients with lung cancer. Regarding this issue, during the last few years there has been a growing interest in molecular biology of lung cancer. As the molecular characteristic of cancer have become better understood, prognostic models have been developed for lung cancer that incorporate biological markers, immunohistochemical properties and genetic features in addition to hystologic subtype, age of patients and extent of disease (TNM-stage). Mutation of the p53 tumor suppressor gene, which is localized on human chromosome 17p13, it has been observed in many human cancers and is the most common mutation in lung cancers [1]. The p53 phosphoprotein, a 53-kD nuclear protein produced by this gene, act**s** in its wild-type conformation as a transcription factor and DNA binding protein, and this activity results on inhibition of cell proliferation by blocking entry into the S-phase of the cell cycle [2]. Mutant p53 proteins lead to the synthesis of stabilized proteins with an half-life increased from 20 min to several hours, compared with wild-type p53, and consequently accumulate in the nucleus to levels easily detectable by immunohistochemestry [3]. Since a first study demonstrated the relevance of p53 immunohistochemical expression in lung cancer [4], several reports have been carried out on the clinical and prognostic significance of p53 alteration in this field, but the results are not always of univocal interpretation, with a few meta-analyses inclining towards abnormal p53 status being associated with poorer prognosis [5,6]. Actually, there are many publications in which p53 overexpression, in small biopsies, obtained by bronchoscopy or transbronchial biopsies, burdens for poor prognosis in advanced non-small cell lung cancer [7-10].

The antibody Ki67, which recognizes a nuclear antigen in proliferating cells, has been widely used to estimate growth fraction in different cancer lesions [11,12]. Despite a large number of studies performed in lung cancer patients, the prognostic value of Ki-67 for survival remains controversial and, till now, there are very few meta-analysis reports on its importance in human lung cancer [13-19].

Aim of the present study is to determine the correlation of p53 protein and Ki67 antigen, immunohistochemically detected in bronchial biopsies, with survival of patients with non small cell lung cancers.

## Methods

### Patients

We studied samples obtained from 19 consecutive patients that had been undergone to bronchoscopy, with diagnostic intent, in our Unit, between January 2008 to December 2010. We used flexible fiberoptic bronchoscopy (BF-1 T40; Olympus; Tokyo, Japan) and all samples were collected by the same operator. Only patients with positive histological diagnosis entered into the study. The clinical staging was defined with the usual method [20]. There were 5 women and 14 men, with ages ranging from 57 to 83 years (mean ± SD, 66.0 ± 6.7). Diagnosis of the 19 non-small cell lung cancer (NSCLC) included: 12 adenocarcinomas, 5 squamous cell carcinomas, and 2 in which the hystological pattern was not clearly identified by the two operators. Thus, in these 2 cases the term of non-small cell lung carcinoma (NSCLC) not otherwise specified (NOS) was used,according to the recent revision of NSCLC in small biopsies [21]. The study follow-up lasted to each patient’s death. Clinicopathological data of all patients are reported in Table 1.

**Table 1:** Characteristics of the patients

**#**	**Name**	**Sex**	**Age**	**Diagnosis**	**Smoking**	**Staging**	**p53**	**Ki67**	**Survival**
1	MS	M	68	1	+	IIIA	+	+	2
2	SF	M	62	1	+	IV	+	+	4
3	MS	M	74	3	+	IIB	+	+	5
4	TP	M	74	2	+	IIB	-	-	10
5	SG	M	72	1	+	IV	+	+	2
6	BS	M	67	1	+	IIA	+	-	14
7	PG	M	57	2	+	IB	+	+	4
8	GP	M	64	2	+	IIIB	-	-	20
9	CS	M	69	1	+	IIIB	+	-	8
10	SS	M	83	1	+	IIIA	-	-	17
11	PG	M	59	1	+	IIA	-	-	21
12	ZF	M	66	1	-	IB	-	+	6
13	RM	F	64	2	-	IIIB	-	-	7
14	TF	F	68	1	+	IIIA	+	+	5
15	MS	F	59	1	+	IIB	-	-	10
16	CR	F	70	2	-	IIA	-	-	9
17	RS	M	59	3	+	IIIA	+	-	5
18	BA	F	64	1	+	IIIB	-	+	7
19	BR	M	58	1	+	IIA	-	-	15

Age at the time of diagnosis; Smoking more than 10 pack/yrs = +; Diagnosis: 1 = Adenocarcinomas, 2 = Squamous Cell, 3 = NSCLC-NOS; Survival: time from the diagnosis (months). For p53 and Ki67 + or – see the test.

### Immunostaining

Immunohistochemical staining was performed on formalin-fixed, paraffin-embedded sections of 5 um with monoclonal antibodies to the p53 protein (DO-7; DAKO, Japan) and Ki67 (Ab297M, Biogenex, U.S.A.). Similar procedures were followed for the usual treatment of deparaffination, with xylene, absolute alcohol and distilled water. Ki-67 and p53 sections were heated in a microwave oven 4 times, each of 5 min, in citrate buffer-solution. The slides, after at least 30 min of air-drying, were incubated with the primary antibodies against p53 and Ki67. Dilutions (raging between 1:25 and 1:50) and time of incubation (30 to 60 min) were depending on the batch of antibody. After washing with Phosphate Buffered Saline (PBS) secondary antimouse antibodies were added (biotinylatedantimouseIgG; Nichirei, Japan) both for Ki-67 or P53. Afterwards the slides were incubated with streptavidin-peroxidase reagent (Nichirei, Japan) and again the time of incubation was depending by the “data-sheet” of the antibody. After another PBS wash, the final staining was provided using a 0,05% solution of diaminobenzidinetetrahydrochloride in PBS usually for 10 min. Finally, sections were counterstained with Meyer’s haematoxylin. For p53 a tumor was considered to be immunopositive when the number of cells with nuclei red-stained exceeded 15% [10], without a semiquantitative classification. Likewise, for Ki67 the “cut-off” point for positivity was considered when >25% positive cells were observed by counting more than 1,500 cancer cells randomly selected throughout each section using a 40x objective [13].

### Data analysis

The Kaplan-Meier method was used to estimate the probability of overall survival as a function of time (starting from the date of diagnostic bronchoscopy to that of death from cancer), and difference in the survival of subgroups of patients were compared with Mantel’s log-rank test. Multivariate analyses were performed using the Cox regression model to study factors that independently influenced overall survival. Statistical significance was set a P value <0.005 and all test were two-tailed as were the reported P values.

## Results

**Figure 1:**
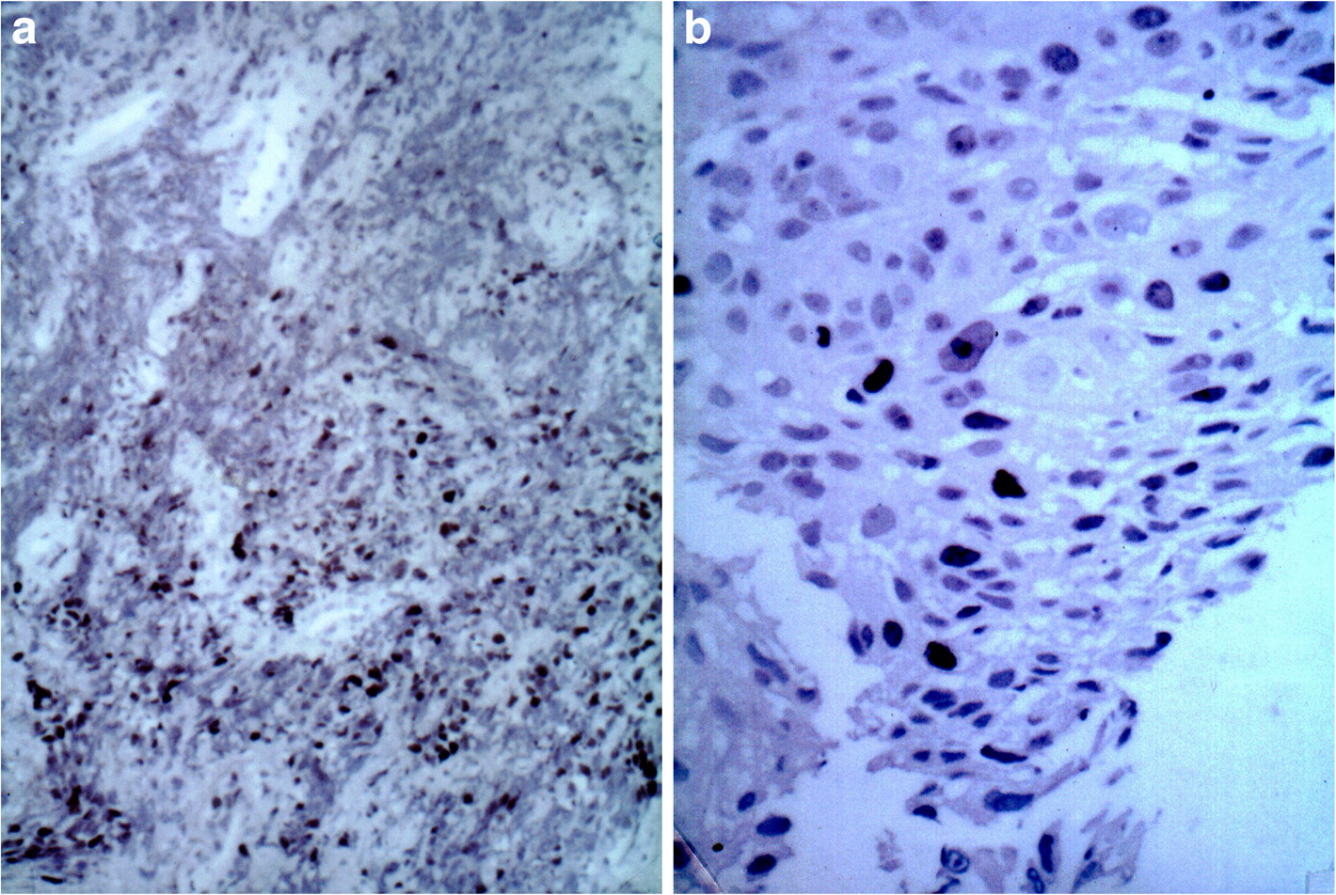
Positive immunostaining for p53, adenocarcinoma of the lung. b: Positive immunostaining for Ki67, squamous cell carcinoma of the lung.

We found p53 reactivity in 9 of the patients (47.37%). The antibody used in this study reacts with wild type and mutant type of the p53 protein and the nuclear reactivity, as expressed before in “Methods” section, was designed to be “positive” (+ve). For Ki67 immunostaining, reactivity between antibody and antigen localised in nuclei of proliferating cells, designed + ve as above indicated, was found in 8 of the patients (42.10%). By contrast we designed as “negative” (−ve) reactivity against p53 antibodies or Ki67 under the “cut-off” points of 15% for p53 and 25% for Ki67. Only tumoral section showed immunoreactivity both for p53 or Ki67, indicating that our immunostaining procedures do not generate false positives. In Figures 1a and 1b we shown photomicrographs of p53 + ve and Ki67 + ve sections of different histotypes are shown.

**Figure 2:**
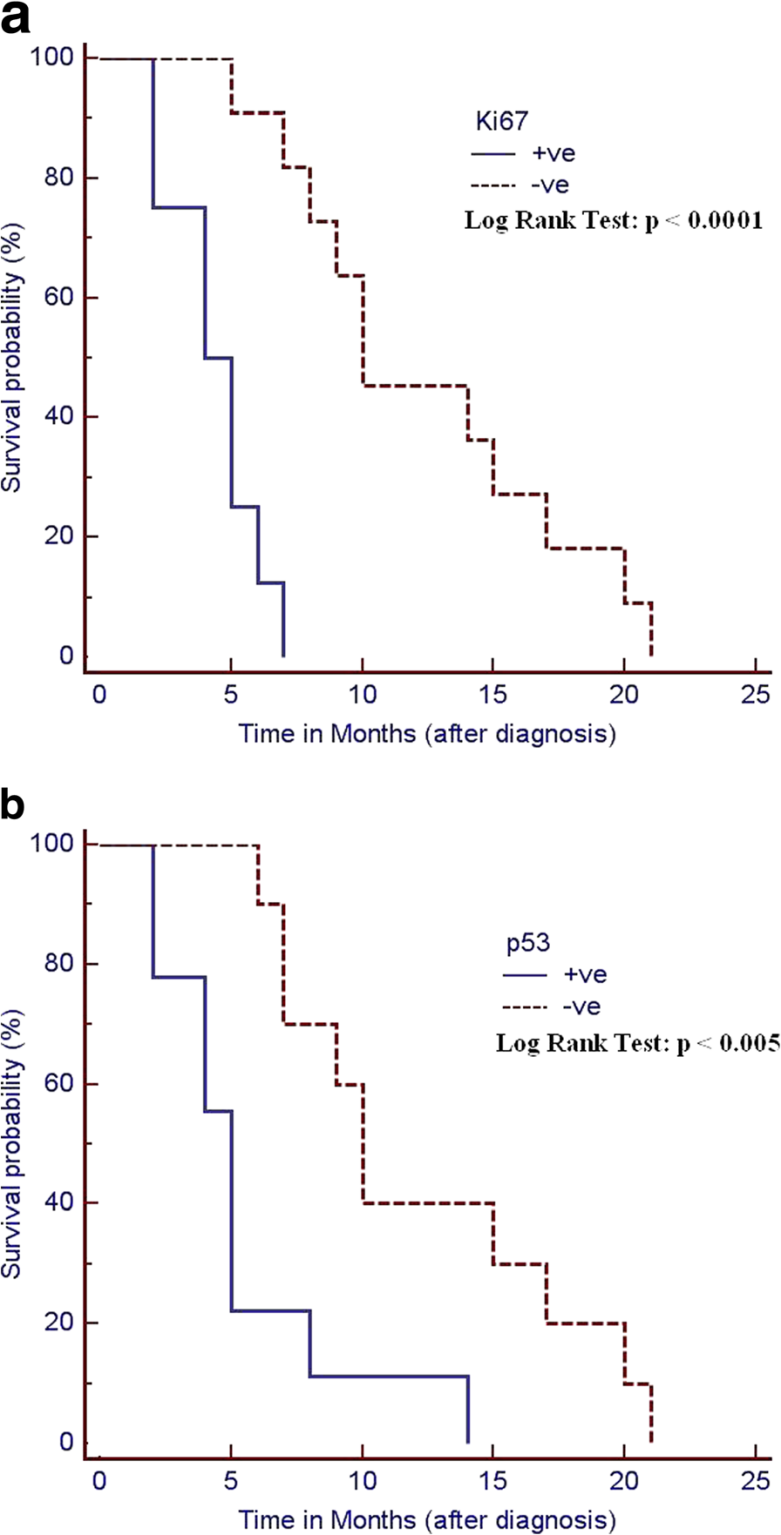
a: Overall-survival of all patients included in the study, with p53 immunohistochemicalresults. b: Overall-survival of all patients included in the study, with Ki67 immunohistochemical results.

We compared Kaplan-Meier survival curves for the p53 and Ki67 + ve groups and p53 and Ki67 –ve groups in all 19 patients with NSCLC (Figures 2a and 2b). We found that both p53 –ve (median survival: 10 months) and Ki67 –ve (median survival: 10 months) patients had a significantly higher survival rates than p53 + ve (log rank test, p < 0.005, median survival 5 months) and Ki67 + ve (log rank test, p < 0,0001, median survival 4.5 months). Multivariate analysis (Cox proportional hazard model) for age, sex, smoking history, TNM stage, and overexpression of p53 and ki67 was performed to examine the interrelationship of possible prognostic factors and survival (Table 2). In all cases, p53 and ki67 overepression were statistically identified as independent prognostic factors predicting poor survival with a relative risk of 15.31 (p < 0.005) and 19.09 (p < 0.005), respectively. The other studied variables did not show a statistical correlation as independent prognostic factor with survival.

**Table 2:** Cox proportional hazard model analysis of survival time

**Covariate**	**Coefficient b**	**SEM**	**P**	**Exp (b)**	**95% CI of Exp(b)**
Age (<65 vs. >65 yrs)	−0,5287	0,7819	0,4989	0,5894	0,1283 to 2,7076
Ki67 (+ve vs. –ve)	2,9491	0,9966	0,003085	19,0890	2,7339 to 133,2877
p53 (+ve vs. –ve)	2,7283	0,9849	0,004904	15,3068	2,2428 to 104,4664
Sex (M vs. F)	−0,3679	0,6455	0,5688	0,6922	0,1966 to 2,4374
Smoking History	−2,3558	1,0740	0,02827	0,0948	0,0117 to 0,7699
(Smoker vs. Not-smokers)					
TNM Stage (I-IIIA vs. IIIB-IV)	0,3726	0,6508	0,5670	1,4515	0,4080 to 5,1642

## Discussion

First histological diagnosis of lung cancer is often obtained by fiberoptic bronchial biopsies. However the utility of extend histological studies with immunohistochemical methods in lung cancer is still debated. We have shown the feasibility of immunohistochemical detection of p53 and Ki67 in bronchial biopsies, performed with diagnostic intent, in patients with histological diagnosis of NSCLC. In our study, the incidence of p53 positivity in NSCLC was 47.34%, and it was 42.1% for Ki67 positivity. Furthermore, utilizing these small biopsy lung specimens we compared p53 and Ki67 positivity with survival rate. There are few studies describing p53 overexpression in samples obtained in a non surgical setting and it relationship to survival rate [7-10,22-24]. Murakami and coll. [23] examined 25 specimens, from bronchial biopsies, of 23 patients, and they were able to detect p53 gene mutations in 10 of 25 (40%) of bronchoscopic biopsy specimens. These authors, who studied altogether 206 specimens from 66 patients obtained with different sampling methods, such as bronchial biopsy, brushing, washing, thoracentesis and percutaneous needle aspiration, concluded that p53 mutation is associated with a poor prognosis in patients with advanced NSCLC. A larger sample of patients was studied by Kawasaki and coll. [9] with transbronchial biopsies. They found 98 p53-positive immunostaining patients from a total of 175 (56%), and regarding NSCLC 61 p53-positive from a total of 111 patients (55%). Also in this study, a significative poor prognosis was found only in patients with stages III/IV and p53 positive immunostaining. Nevertheless, the first study in which p53 positivity was related to poor prognosis was that of Quinland and coll. [25]. They studied 49 NSCLC cases of stage I or II patients surgically-treated, and they found a significative relationship between p53 accumulation and poor prognosis. Their immunohistochemistry method is similar to our, with a monoclonal antibody that recognizes both mutant and wild-type forms of p53 protein. By contrast, subsequent studies not always confirmed results of Quinland and coll. of the relevance of p53 immunostaining in the early stages as a prognostic factor of lung cancer [26-31]. In our study, most patients received only palliative care, or did not complete the chemotherapy (kt) treatment, and only 6 patients were surgically treated. This could be a confusing factor of our statistical analysis. In fact, positive p53 staining seems to be correlated with unresponsiveness to kt in NSCLC [10] and p53 mutation has been shown to determine the sensitivity of cells to anticancer agents [32].

The use of Ki67 immunostainining in lung cancer is not easy to apply and the feasibility of Ki67 in specimens of small size like the bronchial biopsies is still controversial.Scagliottiet all. [14] firstly indicated that patients with a higher Ki67 score (>25% positive cells) at diagnosis had a significantly lower disease-free survival. In the present study, we have chosen a similar “cut-off point”: a percentage of stained tumour cell greater than 25%, and the results were in accordance with other ones regarding feasibility in small size specimens [33]. Afterwards, other studies investigated the role of Ki67 overexpression in NSCLC [15,34] demonstrating that it was associated with unfavourable clinical outcome.

Simultaneous expression of p53 and Ki67 was studied as a prognostic factor in resected lung cancer [16]. Surprisingly, these authors found that combination p53 positivity and low Ki67 expression may represent a favourable prognostic factor. Few years ago, prognostic significance of p53 and Ki67 immunocytochemical expression was studied in surgically treated NSCLC [17]. These authors found a significative statistical correlation only in p53-positive smears in patients of stage 1 and particularly in patients with adenocarcinoma subtype.

## Conclusion

We have shown the utility of p53 and Ki67 immunostaining of bronchial biopsies in NSCLC. Our data indicate that p53 and Ki67 immunostaining of small size specimens obtained during diagnostic procedures with fiberoptic bronchoscopy may be useful in predicting prognosis in patients with NSCLC. The incidence of immunohistochemical positivity in NSCLC was approximately the same as reported in other studies, and, furthermore, no histologically negative samples was stained, thus indicating the validity of our method. However, these results were obtained in a relatively small number of patients, such as we were not able to predict a relationship between positive immunostaining and resistance to chemotherapy.

We believe that a simple, inexpensive method, as that reported in the present study should be useful in predicting prognosis of patients with NSCLC. More studies are needed to understand the possible relationship of positive stain for p53 and Ki67 with the resistance to chemotherapy.

## Competing interest

The authors declare that they have no competing interest.
